# Singlet Fission Luminescent Solar Concentrators

**DOI:** 10.1021/acs.nanolett.5c04255

**Published:** 2025-10-31

**Authors:** Tomi K. Baikie, Jesse Allardice, Simon A. Dowland, Pratyush Ghosh, Aaron Li, James Xiao, Géraud Delport, Ashish Sharma, Neil C. Greenham, Akshay Rao

**Affiliations:** † Cavendish Laboratory, 2152 University of Cambridge, J.J. Thomson Ave, Cambridge CB3 0HE, U.K.; ‡ Cambridge Photon Technology, J.J. Thomson Ave, Cambridge CB3 0HE, U.K.; § Department of Chemistry, Massachusetts Institute of Technology, Cambridge, Massachusetts 02139, United States; ∥ CNRS, Institut Photovoltaïque d’Ile de France (IPVF) UMR 9006, 18 Boulevard Thomas Gobert, 91120 Palaiseau, France

**Keywords:** Singlet Fission (SF), Photon Multiplication (PM), Luminescent Solar Concentrator (LSC)

## Abstract

Luminescent solar concentrators (LSCs) present a promising avenue for solar energy harvesting, utilizing transparent matrices embedded with light-absorbing chromophores to concentrate incident solar radiation. Photon-multiplier luminescent solar concentrators (PM-LSCs) contain chromophores boasting over 100% photoluminescence quantum efficiency. Although PM-LSCs may bypass free energy losses observed in traditional LSC systems, experimental PM-LSCs have exhibited optical efficiency sensitivity to photon flux. Here, we demonstrate a PM-LSC utilizing singlet fission (SF), an exciton multiplication process. We apply large-area films of absorbing TIPS-tetracene mixed with tetracene-carboxylic acid-ligated PbS quantum dots and demonstrate they are suitable for solid-state LSC devices. We find that although SF-LSCs present pathways to mitigate fluence limitations observed in quantum cutting systems, challenges persist due to triplet–triplet annihilation (TTA) at higher photon fluxes. The potential of SF-LSCs to overcome fluence limitations in PM-LSCs suggests a promising avenue for future development.

## Graphical abstract

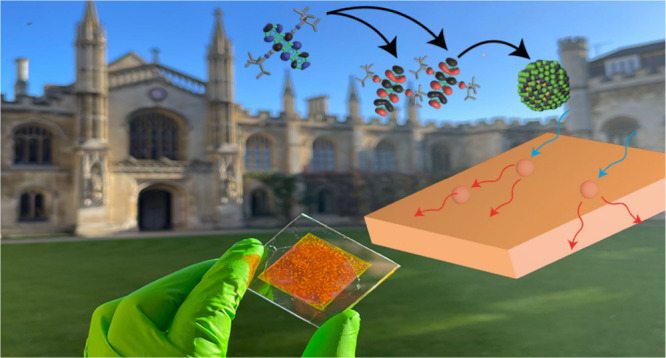

Luminescent solar concentrators (LSCs) represent a promising avenue in solar energy harvesting by concentrating incident solar radiation. These devices are constructed using a transparent matrix, typically composed of plastic or glass, coupled with light-absorbing chromophores. As in Figure 1A, chromophores absorb incoming sunlight and subsequently reemit it. The emitted light is then confined within the matrix leading to its concentration. The concentrated light is subsequently harvested by photovoltaic (PV) strips placed at the periphery or on the surface of the LSC. Usefully, LSCs can mitigate the impact of optical shading on PV cells and can selectively downshift the solar spectrum, aligning it with the optimal spectral characteristics for specific applications.^1^ This adaptability renders LSCs versatile and suitable for a broad range of applications, spanning from agriphotovoltaics,^2–5^ reaction chemistry,^6–10^ building integrated photovoltaics^11,12^ and solar-pumped lasers.^6^

**Figure 1:**
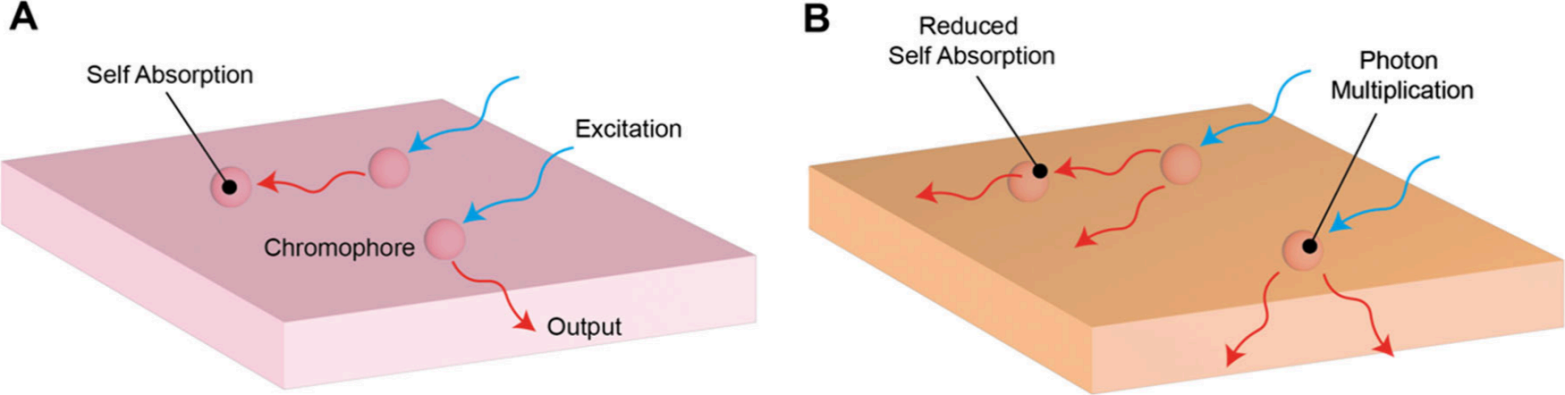
A, Schematic of operation of a luminescent solar concentrator (LSC). Photons are absorbed by chromophores that reemit light at a longer wavelength. The redder photons are confined within the device, resulting in a concentrated beam impinging on photovoltaics mounted on the edge of the LSC. A major loss pathway in the LSC is photon reabsorption. B, In an optimal PM-LSC two photons are emitted for every absorbed incoming photon. As the wavelength shift is inherently large a PM-LSC device should exhibit negligible self-absorption.

Photon-multiplier luminescent solar concentrators (PM-LSCs), depicted in Figure 1B, contain chromophores that exceed 100% photoluminescence quantum efficiency.^13–15^ These technologies offer an advantage in radically reducing self-absorption losses relative to the Stokes shift of traditional dyes. In many dyes, the emission and absorption spectra overlap and therefore emitted photons in LSC may be reabsorbed which gives rise to additional nonradiative decay pathways or surface losses from the LSC, which in turn constrains the geometry and efficiency of the device. The nature of PM-LSC induces negligible overlap between absorption and emission, lessening the photon reabsorption within the LSC.

Thermalization losses in a solar cell refer to the energy lost when high-energy photons generate charge carriers with excess energy, which is dissipated through phonon interactions rather than being converted into electrical power. PM-LSCs coupled to silicon photovoltaics can drastically reduce thermalization losses, as PM-LSCs are able to convert this thermalization loss into useful electricity.^13,14,16–18^

PM-LSCs also offer more subtle advantages. Traditional LSCs exchange photon energy for entropy to fulfill the thermodynamical requirements for concentration.^19^ This inherent energy loss is given by the difference in the energy between the absorbed photon and the emitted one. However, PM-LSCs may concentrate light through entropy generation arising from the generation of multiple photons, meaning, unlike traditional LSCs, there need not be any photon energy loss to give rise to concentration.^20^ This offers a significant benefit in the context of solar energy capture.

The concentration ratio for any LSC should be tailored such that the photon flux impinging on the coupled solar cells of the device is within goldilocks region of increased efficiency of silicon devices, but before Auger and other flux-dependent recombination pathways of the photovoltaic dominate.^16^ For a PM-LSC this is likely to be lateral size around 10 by 10 cm, hence extremely large devices are unlikely to be useful in the context of LSCs designed to capture solar energy using photovoltaics.^16^

Existing demonstrations of PM-LSCs consist of perovskite crystals doped with Yb^3+^ impurity ions.^13,14^ It has been argued that Yb^3+^ doping in perovskites introduces defects that rapidly localize excitation energy in the vicinity of the Yb^3+^ site. In or around this defect, the formation of the trapped excited state is followed by nearly resonant energy transfer to form two excited Yb^3+^ ions. This process is referred to as quantum cutting.^13,14^ The lifetime of Yb^3+^ emission is close to the radiative limit,^21^ with reported PLQEs above 100%. We note that there has been considerable debate in the community on the reproducibility of these systems and that there has been, until date, no demonstration of a PV device with EQE above 100%, despite these systems being reported a number of years ago. Although LSCs based on quantum cutting meet many criteria for PM-LSCs, a key challenge is that efficiency of the quantum cutting process is significantly reduced with increasing photon flux. This arises as ytterbium-doped perovskite crystals exhibit long-lived excited states with lifetimes on the order of milliseconds. If an excited state cannot quickly recover to a photon-accepting state, photons are lost to either nonradiative decay channels or lower-efficiency radiative channels. As such, existing PM-LSC demonstrations exhibit power conversion efficiencies much below those of nanocrystals or dyes at terrestrial photon fluxes.^13–15^

Singlet fission (SF) is an exciton multiplication process that occurs in organic semiconductors, where the initially photogenerated singlet exciton may convert to an entangled triplet pair, which then breaks up to form two free triplet excitons.^17^ SF-based PM-LSCs hold promise as they might overcome fluence limitations observed in conventional PM-LSCs. We have previously shown that SF can be exploited to achieve photon multiplication by harvesting triplets of the SF chromophore 5,12-bis­((triisopropylsilyl)­ethynyl)­tetracene), referred to as TIPS-Tc, through lead sulfide (PbS) quantum dots.^22^ This harvesting is possible when PbS quantum dots covered with 6,11-bis­((triisopropylsilyl)­ethynyl) tetracene-2-carboxylic acid)) ligands, referred to as PbS-Tet-CA, are used to facilitate triplet energy transfer.

Herein, as shown in Figure 2, we utilized a film of TIPS-Tc and varying amounts of PbS-Tet-CA quantum dots (see Methods 1 for details, SI Section 1 for concentration optimization and Figure 2C for absorption and PL measurements) to develop an SF-based PM-LSC. As depicted in Figure 2D, singlets are initially generated in TIPS-Tc and undergo SF to from triplets which then diffuse until they encounter a PbS-Tet-CA nanocrystal into which their energy can be transferred.^23–25^ Radiative recombination then occurs, with each triplet transferred to the dot resulting in the emission of a low-energy photon, which can then be directed by the LSC to PV attached to the edges of the LSC. Similar SF processes have been demonstrated to enhance photocurrent in organic solar cells, organic/nanocrystal hybrid solar cells, and tandem silicon solar cells.^26–29^

**Figure 2:**
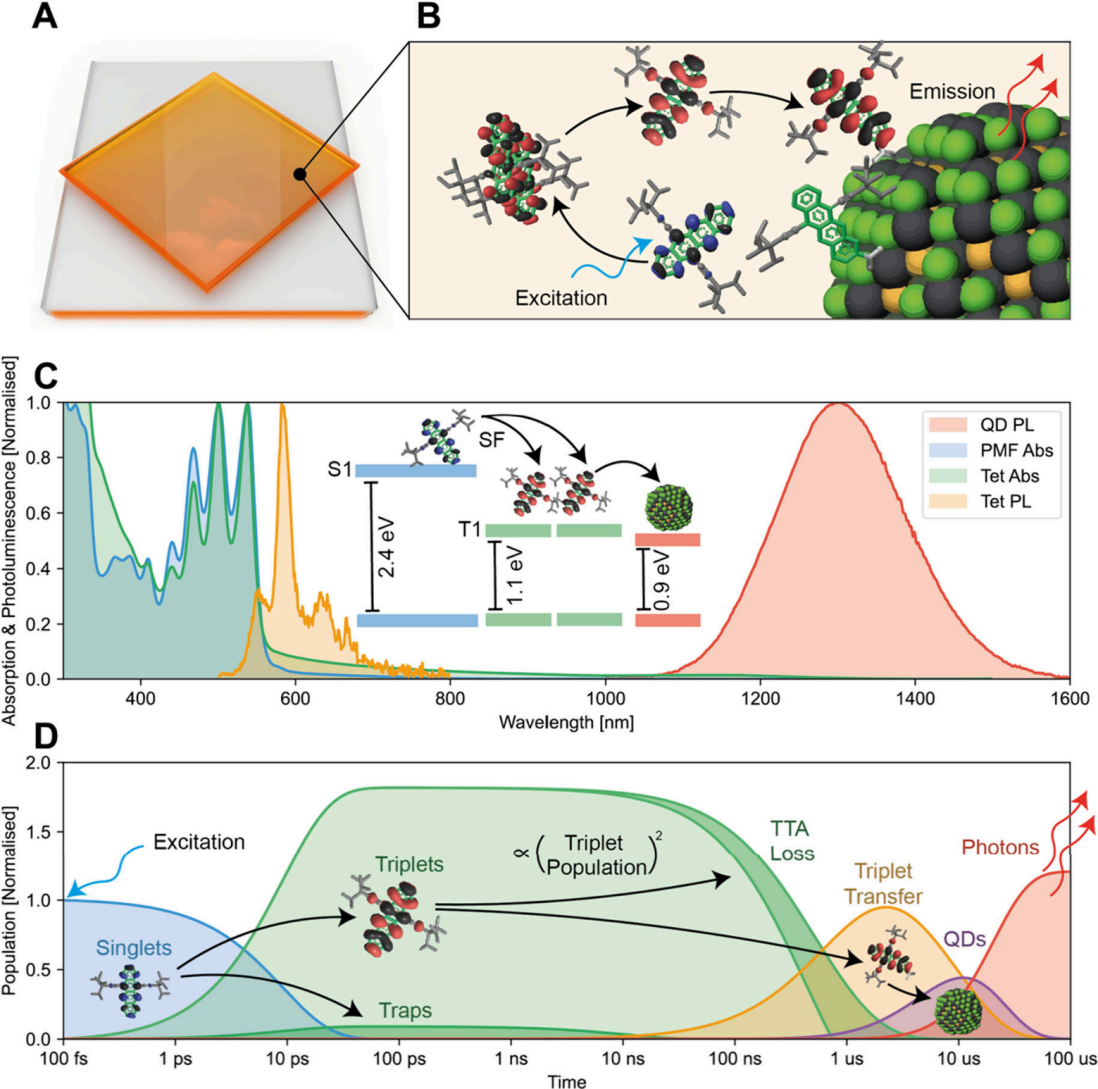
A, PM-LSC schematic depicting a substrate with high refractive index that allows outcoupling of a beam of concentrated photons from the substrate edges. The photons arise from, B, a film of photomultiplier material. The film consists of TIPS-Tc which undergoes efficient singlet fission upon excitation, whereafter the triplets then diffuse to PbS-TIPS-Tc-CA where they emit. C, Photoluminescence (PL) and absorption spectra (Abs) of the TIPS-TC film and the PbS-TIPS-Tc-CA photomultiplier film (PMF). D, Population dynamics of this process; lifetime references are given in SI Section 1. A high-energy photon is absorbed, forming a singlet state in the TIPS-Tc, which then undergoes singlet fission. Some singlets and triplets are lost to traps. Some of the resulting triplets undergo triplet–triplet annihilation (TTA), a process that is a quadratic function of the triplet population. The remaining triplets transfer to emissive quantum dots through TIPS-Tc-CA.

The quantum efficiency of a singlet fission photon multiplication process η_PM_ may be understood by the quantum efficiencies of four steps; the efficiency of SF, η_SF_, the efficiency of triplet diffusion from the SF material to the vicinity of the QD, η_TD_, the efficiency of triplet transfer from the SF material into the QDs, η_TT_, and the efficiency of light emission from the QDs, η_PL_,

1 $$\eta_{PM}=\eta_{SF}\eta_{TD}\eta_{TT}\eta_{PL}$$

η_SF_ is known to be highly sensitive to molecular packing and the presence of the nanocrystal is known to disrupt the packing of the molecules over which the singlet fission takes place.^24,25,30^ As such, control of the morphology is crucial to achieve optimal performance of an SF-based PM-LSC. For films of TIPS-Tc mixed with PbS-Tet-CA, SF efficiency is optimized when QDs are well dispersed within small TIPS-Tc crystallites.^31^ The parameter η_TD_ is controlled by the triplet diffusion rate as triplet excitons need to diffuse close to the nanocrystal to undergo energy transfer via a short-range Dexter-type process.^32^ Triplet diffusion rates are very high in crystalline films of tetracene,^33^ so triplet diffusion to QDs can be efficient near crystalline TIPS-Tc domains.

To achieve efficient transfer to QDs (high η_TT_), such as in a PM-LSC, the energy of the QD must lie below the triplet energy of the SF chromophore, yet it must also be above the silicon bandgap for photovoltaic operation. This remains a challenge in experimentally demonstrated systems, as it does in ours, where the emission from the PbS QD indicates the bandgap (approximately 0.9 eV, see Figure 2C), is below that of silicon (approximately 1.1 eV). Although it is relatively easy to systematically alter the bandgap of the QD, it is not trivial to identify higher-energy molecules that will undergo efficient SF, which limits the extent to which the QD emission energy can be raised.

Harvesting SF-generated triplet excitons has been previously demonstrated in solution, with relatively homogeneous spatial distributions of absorbers and emitters.^25,34,35^ Although solution based LSCs are possible,^36^ they give rise to practical challenges and as such are rarely produced in lab environments and result in longer time scales in triplet formation.^37^ To fabricate efficient SF-based PM-LSCs, we follow the QD surface engineering approach reported by Allardice et al. to achieve a film morphology where QDs are well dispersed within small crystallites of TIPS-Tc.^38^ Here, PbS QDs are covered with the Tet-CA ligand that is structurally similar to the SF host, TIPS-Tc. The SF yield of films fabricated through this approach is high, η_SF_ = 190%, followed by very efficient triplet energy transfer to the QDs, η_TT_η_TD_= 97 ± 11%, giving rise to a ∼ 190% exciton multiplication factor.^38^ However, the maximum η_PL_of the QDs used within this work are low, typically around η_PL_≈30% in toluene.^38^ Higher η_PL_ efficiencies can be achieved for lower emission wavelengths.

Films here are generated through a blade coating method, with optimized 50% mass fraction QD loading (see SI Section 1) that may give rise to surface inhomogeneity on the surface of LSC. To understand how this might affect yields we performed time-resolved microscopy on the samples (see Methods 3). We determined the short-lifetime (0–3 ns) fluorescence and a long-lifetime (100–500 ns) fluorescence for each pixel between 550 ± 40 nm which corresponds to singlet excited state of TIPS-Tc. We assign the initial fluorescence to direct photoexcitation and the delayed component to the repopulation of singlet states from TTA. Therefore, Figure 3A and Figure 3B are a map of TTA in the TIPS-Tc film and TIPS-Tc with QDs film, respectively. In the pristine film, we observe a uniform triplet density across the film. However, for the sample including the dots, fluorescence is clumped into submicron regions, where we suppose triplets are more strongly able to diffuse to dots (in more detail in SI Section 2). We determined the efficiency of SF of a wide area of the film to average out these inhomogeneities and found the overall PLQE of the film to be 17 ± 2% (see Methods 6 for details).

**Figure 3:**
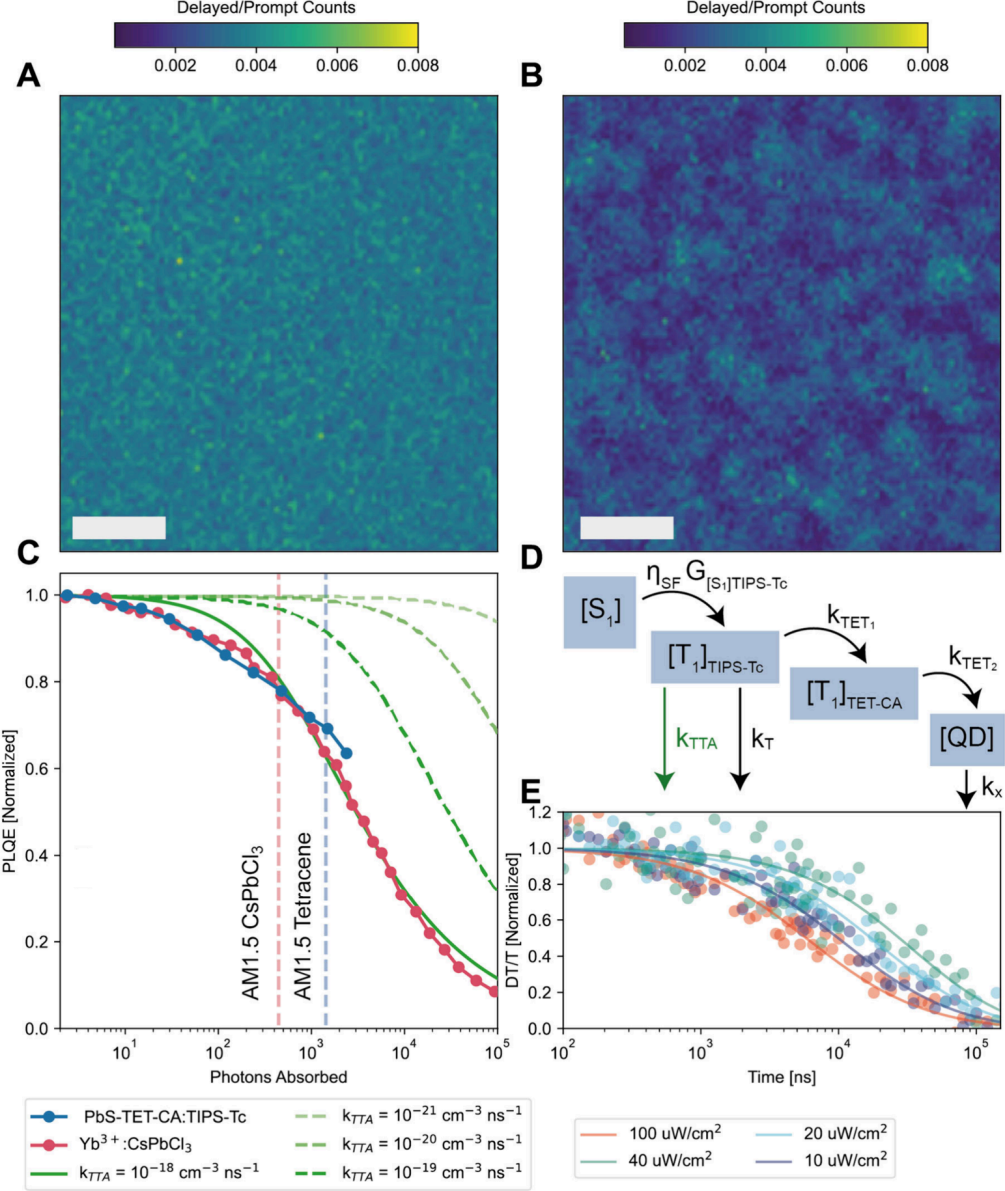
PLQE and TA. PL maps of A, TIPS-Tc, and B, PbS-TET-CA:TIPS-Tc films. Scale bar is 5 μm. The TIPS-Tc PL decay is detected at 550 ± 40 nm after 405 nm excitation at a fluence of 50 μJ cm^–2^ and 2 MHz pump repetition rate. The delayed (100–500 ns) PL is normalized by the prompt PL counts (0–3 ns). C, Normalized PLQE under steady state illumination from the PbS-TET-CA:TIPS-TC system (in blue) described here as a function of absorbed photons and from a lanthanide system (in red) previously reported by Erickson et al.^15^ Modeled TTA rates impacting PL efficiencies are given in green for 4 different rates. Vertical dashed lines indicate the number of photons absorbed at terrestrial solar fluence. D, Modeled reaction scheme, highlighting k_TTA_ loss. E, Normalized nsTA kinetics at 970 nm corresponding to TIPS-Tc triplet excitons under 20, 40, 80, and 200 uJ cm^–2^ per pulse at 1 kHz, corresponding to powers of 10, 20, 40, and 100 μW cm^–2^, respectively. The solid lines fit the data and illustrate the nonlinear behavior of the triplet population dynamics, indicating the presence of the TTA loss pathway.

To test for the effect of fluence on the photon multiplication process, we measured the PLQE of PbS-TetCA:TIPS-Tc films at different excitation fluences. As shown in Figure 3C, the normalized PLQE yield dropped significantly as a function of excitation fluence. Using nanosecond transient absorption measurements, we observe that the lifetime of triplet excitons in the films decreases with an increase in fluence (Figure 3E), consistent with our theory that triplet–triplet annihilation (TTA) is the dominant decay pathway of SF-generated triplet excitons. The loss of triplets to TTA will decrease the efficiency of triplet transfer to QDs, resulting in a lower PLQE with increasing fluence. To examine the effect of TTA on the PLQEs quantitatively, we model the PLQE of the films as a function of the excitation fluence (Figure 3D). Eqs 1 - [Disp-formula eq3] outline the proposed reaction scheme for the system,

2 $$\left(k_{T}+k_{TET_{1}}\right){\left[T_{1}\right]}_{TIPS-Tc}+k_{TTA}{\left({\left[T_{1}\right]}_{TIPS-Tc}\right)}^{2}=\eta_{SF}G_{{\left[S_{1}\right]}_{TIPS-Tc}}$$

3 $$-\left(k_{TET_{2}}\right){\left[T_{1}\right]}_{TET-CA}+k_{TET_{1}}{\left[T_{1}\right]}_{TIPS-Tc}=0$$

4 $$-\left(k_{X}\right)\left[QD\right]+\left(k_{TET_{2}}\right){\left[T_{1}\right]}_{TET-CA}=0$$

where, $\eta_{SF}G_{{\left[S_{1}\right]}_{TIPS-Tc}}$ is the generation rate of triplet excitons in TIPS-Tc, *k*_*T*_ is the rate of decay of *T*_1_ excitons to the ground state of TIPS-Tc, $k_{TET_{1}}$ is the rate of triplet energy transfer from TIPS-Tc to Tet-CA ligands, $k_{TET_{2}}$ the rate of energy transfer from Tet-CA ligands to the PbS dots, *k*_*TTA*_ is the triplet–triplet annihilation rate which competes with triplet energy transfer to the dots, *k*_*X*_ is the inverse of the photoluminescence lifetime of PbS-Tet-CA QDs and square brackets denote respective populations. Triplet–triplet annihilation generally gives rise to the formation of a triplet or a singlet, TTA-T or TTA-S, respectively. Here we explicitly assume that *k*_*TTA*_ describes an annihilation process that is not able to regenerate the singlet [*S*_1_] (or single triplet [*T*_1_]) state described in eqs 1–3 (or equivalently Figure 3D) and instead returns to the ground state. This means that regeneration of the excited state is not possible through *k*_*TTA*_, and it describes a ${\left[T_{1}\right]}_{TIPS-Tc}$ population dependent loss.

The values of the parameters *k*_*T*_, $k_{TET_{1}}$ , $k_{TET_{2}}$ and *k*_*X*_ chosen to simulate the traces in Figure 3C are 0.01 μs^–1^, 0.3 μs^–1^, 1.6 μs^–1^ and 2.5 μs^–1^, respectively, and determined from similar PbS-Tet-CA and TIPS-Tc blends reported previously.^38^ The decrease of PLQE with fluence of PbS-Tet-CA and TIPS-Tc blend films is explained by the fluence-dependent nonradiative annihilation of triplet excitons in the films (*k*_*TTA*_= 10^–18^cm^–3^ns^–1^, see dark green line in Figure 3C). This value of *k*_*TTA*_ value is similar to the rate of TTA in closely packed films of acenes.^39^ The significant effect of *k*_*TTA*_ on photon multiplication performance suggests that minimizing losses due to TTA is a crucial parameter in the design of SF-based PM-LSCs. Importantly, reduced *k*_*TTA*_ rates (dashed green lines in Figure 3C) may guide future SF systems; specifically in this system *k*_*TTA*_= 10^–20^cm^–3^ns^–1^ or lower would suggest negligible optical efficiency depreciation in the terrestrial regime.

Surprisingly, the steady state PLQE yield as a function of photons absorbed closely followed the trends set by the optimized quantum cutting system described by Erickson et al.^15^ (see Figure 3C, in red). Due to the larger TIPS-Tc absorbance relative to the Yb^3+^ absorbance under standard terrestrial solar irradiation, the SF system has a larger efficiency depreciation when operating at solar intensity relative to the quantum cutting system, see vertical dashed lines in Figure 3C. However, the SF system described here also offers a broader absorbance, improving the efficiency relative to the quantum cutting system in a terrestrial environment, as more photons may undergo a photon multiplication process relative to the quantum cutting system.

We coated the SF system as larger films on high refractive index substrates, to generate 5 cm × 5 cm devices (see Figure 4A). Using spatially resolved photoluminescence techniques in an integrating sphere (see Figure 4B and Methods 6) we determined the effective path length^41^ of photons within the LSC. We recorded an almost negligible photon loss of less than 0.1% cm^–1^ – (see Figure 4B, inset), highlighting the strengths of the SF system in overcoming the major loss channel of photon reabsorption in LSCs. We assign the majority of this loss to scattering. The optical efficiency was determined through a previously reported model^41^ utilizing the above photon loss value and PLQE measurements of the chromophore, yielding ∼ 13% at low fluence. The theoretical PCE under standard terrestrial illumination conditions is extremely poor – < 0.01% - as most of the photons from the photoluminescence spectrum fall below the silicon PV absorption. In Figure 4C, and adapting eqs 1 - [Disp-formula eq3] to the steady state measurement, we outline the impacts of improving *k*_*TTA*_ and in the blueshift of the QD. The SF-LSC has remarkable potential in improving upon the current state of the art if the photoluminescence can be brought to overlap with the solar cell EQE and the *k*_*TTA*_ improving by an order of magnitude.

**Figure 4:**
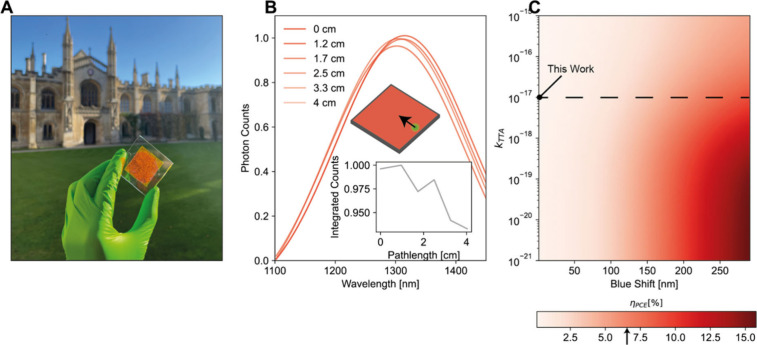
LSC details. A, Photograph of 5 cm × 5 cm LSC. B, Fitted spatially resolved photoluminescence spectra (with subtended angle correction), highlighting minimal reabsorption of the photoluminescence over the length of the LSC. Inset, integrated counts normalized to the maximum value as a function of photon path length. C, Simulated power conversion efficiency as a function of k_*TTA*_ and QD blueshift keeping all other parameters fixed (the QD PLQE remains at 30%). The black arrow on the color bar indicates best reported LSC PCE.^40^

Our central conclusion is that for SF-LSCs operating effectively in the terrestrial environment requires mitigation of the TTA loss pathway in a SF system. Expanding on our previous assumption, where *k*_*TTA*_ describes a loss to the ground state, TTA generally produces a singlet (through TTA-S) or a triplet state (through TTA-T);

5 $$\begin{array}{l} TTA-S:,T+T\to S^{n}+S_{0}\\ TTA-T:,T+T\to T^{n}+S_{0} \end{array}$$

Ideally, a material that exhibits strong TTA-S, i.e. a material is both a good upconverter and photon multiplier, would minimize TTA loss by regenerating a state where SF is again possible. However, this is practically challenging. Spin-statistical constraints and other nonradiative decay processes limit the efficiency of TTA-S pathway to be of the order of ∼ 1% in most solid-state SF materials.^42^

TTA-T we associate with either geminate or nongeminate recombination. Geminate TTA-T, from the initial triplet pair, could be mitigated though symmetry breaking as demonstrated in rubrene systems,^43^ where morphology changes and intermolecular packing preferentially suppresses TTA-T or SF. Nongeminate TTA, associated with the long lifetime loss observed in Figure 3E, is predominately determined by the interaction properties of two triplet states. In our system, if we overcame the clumping present in Figure 3B and introduced a uniform distribution of QDs every few nanometres to reduce the lifetimes of the triplets it would reduce the lifetime of ${\left[T_{1}\right]}_{TIPS-Tc}$ but the quantum dot’s FRET radius would likely result in the singlet transferring directly to the QD, reducing overall efficiency. Analogously to photosynthetic systems, we suppose this could be mitigated by funnelling triplets into another acceptor acting as triplet transport matrix moving the triplet population away from TIPS-Tc toward the PbS QDs. For example, Campos et al. demonstrate efficient SF and triplet funnelling in pentacene-tetracene-pentacene oligomers^44^ and block copolymers,^45^ featuring an ‘energetic cleft’ that promotes the migration of SF generated triplets to low energy sites, thus preventing nonradiative recombination loss. In the design of SF based PM-LSCs discussed here, incorporating a triplet transporter matrix, could present a viable pathway to overcoming the TTA limitations that inhibit SF-LSCs in terrestrial environments.

The chromophore system described here demonstrates solid state SF-LSCs as a promising architecture for harnessing the SF process, although the system used has mismatched energetics in photon emission for silicon photovoltaics. Significantly, the experimental verification of the SF-LSC shows the same strong dependence on fluence as shown by previously demonstrated quantum cutting LSCs. The work suggests that triplet–triplet annihilation (TTA) is the limiting factor in the SF-LSCs and outlines some approaches to overcome these limits.

### 1 Quantum Dot Synthesis

The colloidal PbS nanocrystals capped with oleic acid ligands were synthesized according to a modified method reported by Hines and Scholes.^46^ First, 0.625 g (2.8 mmol) lead oxide (PbO, 99.999%, Alfa Aesar), and 8 mL (25.2 mmol) oleic acid (OA, 90%, Sigma-Aldrich) in 25 mL (78 mmol) 1-octadecene (ODE, 90%, Sigma-Aldrich) was heated in a three-necked round-bottomed flask at 110 °C under vacuum (<10^–2^ mbar), forming a colorless solution. The reaction flask was flushed with nitrogen and heated up to 115 °C and the heating mantle removed. Once the temperature reached 115 °C, a combination of 13.9 mL degassed ODE and 296 μL hexamethyldisilathiane (2.8 mmol, Sigma-Aldrich) was rapidly injected into the lead oleate solution. The flask was allowed to cool naturally to 60 °C and the whole reaction mixture was transferred to an argon glovebox. The size of the PbS nanocrystals was tuned by adjusting the oleic acid concentration. To isolate the PbS-OA nanocrystals from the reaction mixture, ethanol/butanol was added followed by centrifugation at 12000 g. The precipitated nanocrystals were redispersed in toluene. The precipitation and redispersion was repeated multiple times to remove excess reaction solvents and unreacted precursors. The purified PbS-OA nanocrystals were stored in an argon glovebox at a concentration of 20 mg/mL.

### 2 Absorption Measurements

UV–vis-NIR absorption spectra were measured using a Shimadzu UV-3600Plus dual beam spectrometer. The diluted solution samples were measured using quartz cuvettes (Hellma) with a reference sample of neat solvent.

### 3 Time Resolved Microscopy

Samples were excited with a pulsed supercontinuum laser (Fianum Whitelase SC-400-4, 6 ps pulse length) at 0.2 MHz repetition rate. The pump wavelength set to either 535 or 650 nm (full-width at half-maximum 10 nm) with dielectric filters (Thorlabs). Pump scatter from the laser excitation within the photoluminescence path to the detector was filtered-out with an absorptive 900 nm long-pass filter (Thorlabs). The infrared photoluminescence was focused and detected by a single-photon avalanche photodiode based on InGaAs/InP (MPD-InGaAs-SPAD).

### 4 Transient Absorption Measurements

The longtime (ns-μs) transient absorption setup has been described previously.^22^ In short, the pump–probe setup consists of a probe from a LEUKOS Disco 1 UV supercontinuum laser (STM-1-UV, 1 kHz) and a pump generated in a TOPAS optical amplifier, pumped with the output from a SpectraPhysics Solstice Ace Ti:sapphire amplifier (1 kHz). The probe beam is split into a reference and probe and both are focused onto the sample. A pair of line image sensors (Hamamatsu, G11608) mounted on a spectrograph (Andor Solis, Shamrock SR-303i) is used to detect the signal, using a custom-built board from Stresing Entwickslungsburo to read out the signal.

### 5 Spatially Resolved Photoluminescence

The integrating sphere and spatially resolved measurement has been described previously.^41^ Briefly, the LSC described in the main text has 3 edges covered within an integrating sphere. A continuous wave laser beam is scanned across the surface, with the edge output from the uncovered edge recorded using a liquid-nitrogen-cooled InGaAs detector (Princeton Instruments, OMA V). Accounting for the average photon path length and solid angle, the photon output as a function of illumination from the edge can be determined, along with optical efficiency and the theoretical optimum PCE, since the EQE of silicon is known.

### 6 PLQE

The integrating sphere and PLQE measurement procedure has been described previously.^22^ In summary, an integrating sphere with a Spectralon coated interior (Newport 819C-SL-5.3) was used. 515 nm (2.9 × 10^15^ photons per second per centimeter squared at the sample) and 658 nm (1.8 × 10^16^photons per second per centimeter squared at the sample) continuous wave laser diodes (Thorlabs, L515A1 & LP660-SF60) with a beam diameter at the sample of 3 mm was used as the excitation source. Light from the sphere was coupled into an Andor Kymera 328i Spectrograph equipped with an InGaAs detector (Andor, iDus InGaAs 490).

### 7 Blade Coating

Solutions of TIPS Tc and QDs were prepared in dry toluene. Solutions were then blade coated, under nitrogen, onto precleaned (sonicated in DI water+Decon90, DI water, Acetone, IPA) glass substrates at room temperature using an RK (K101) Coater. The speed selected was “speed 8” which is approximately 8 mm per second.

## Supplementary Material



## Data Availability

The data underlying all figures in this article are publicly available from the University of Cambridge repository (https://doi.org/10.17863/CAM.122654).

## Notes

Additional experimental details, modeling, and parameter details (PDF)
