# Supplementary material for: Singlet Fission Luminescent Solar Concentrators
Source: Nano Lett. 2025 Oct 31;25(45):16204–11. doi: 10.1021/acs.nanolett.5c04255 (PMC12616784; doi:10.1021/acs.nanolett.5c04255)
Supplement: Supplementary file 1 [file nl5c04255_si_001.pdf]

Supplementary Information for

Singlet Fission Luminescent Solar Concentrators

Tomi K. Baikie[1], Jesse Allardice[1], Simon A. Dowland[2], Pratyush Ghosh[1], Aaron Li[3], James Xiao[1], Géraud Delport[4], Ashish Sharma[1], Neil C. Greenham[1*], Akshay Rao[1*]

[1]Cavendish Laboratory, University of Cambridge, J.J. Thomson Ave, Cambridge, UK
[2]Cambridge Photon Technology, Cambridge, Cambridge, UK
[3]Department of Electrical Engineering and Computer Science, Massachusetts Institute of Technology, Cambridge, USA
[4]CNRS, Institut Photovoltaïque d'Ile de France (IPVF) UMR 9006 18 Boulevard Thomas Gobert, 91120 Palaiseau, France

Table of Contents

1. Figure 2 Explanatory Note 2
2. Reaction Scheme Motivation 3
3. Optimum Concentration 5
4. Further Analysis 6
Bibliography 13

1. Figure 2 Explanatory Note

This section details the approach taken to generate the graph in **Figure 2D**. The figure is intended as a cartoon schematic and is generated using literature values for *solution* systems. We detail here the values used. $[\mathrm{Trap}]$ and $[\mathrm{TTA}]$ to refer to time dependent trap and TTA losses and all symbols have the same meaning as in the main text. Note that $[\mathrm{Trap}]$ and $[\mathrm{TTA}]$ are not physical populations, but rather a measure of the collective loss of a population passing through these channels.

$$\frac{d[\mathrm{S}]}{dt} = -\,k_{\mathrm{SF}}[\mathrm{S}] - k_{\mathrm{Trap}}[\mathrm{S}]$$

$$\frac{d[\mathrm{Trap}]}{dt} = k_{\mathrm{Trap}}[\mathrm{S}] - k_{\mathrm{Trap,Decay}}[\mathrm{Trap}]$$

$$\frac{d[T_1]_{TIPS-Tc}}{dt} = 2\,k_{\mathrm{SF}}[\mathrm{S}] - k_{TET_1}[T_1]_{TIPS-Tc} - k_{\mathrm{TTA}}[T_1]_{TIPS-Tc}{}^2$$

$$\frac{d[\mathrm{TTA}]}{dt} = k_{\mathrm{TTA}}[T_1]_{TIPS-Tc}{}^2$$

$$\frac{d[T_1]_{TIPS-Tc}}{dt} = k_{TET_1}[T_1]_{TIPS-Tc} - k_{TET_2}[T_1]_{TIPS-Tc}$$

$$\frac{d[\mathrm{QD}]}{dt} = k_{TET_2}[T_1]_{TIPS-Tc} - k_{\mathrm{QD}}[\mathrm{QD}]$$

SI Equations 1-6

Where, for demonstrative impact in **Figure 2D**, at $t = 0$ only $[S] = 1$ and all other populations begin at 0. In steady state operation under terrestrial conditions, $[\mathrm{QD}]$ and $[T_1]$ would not necessarily start at zero. The rates used for the cartoon are given in **SI Table 1** and code to reproduce the figure is available at GitHub depository (Tb8854/SF-LSC-Paper) and DOI (insert in proof).

	**Rate** $[s]^{-1}$	**Reference Lifetime**
$\boldsymbol{k_{\mathrm{SF}}}$	10^{11}	90 ps [1]
$\boldsymbol{k_{\mathrm{Trap}}}$	10^{10}	400 ps [1]
$\boldsymbol{k_{\mathrm{Trap,Decay}}}$	10^{8}	6 ns [1]
$\boldsymbol{k_{TET_1}}$	10^{6}	1.8us[2] , 1 us [3]
$\boldsymbol{k_{TET_2}}$	10^{5}	5.1 us [2] , 92 us [3]
$\boldsymbol{k_{\mathrm{QD}}}$	10^{5}	53 us [3]
$\boldsymbol{k_{\mathrm{TTA}}}$	$0.8\ 10^{6}$	$10^{-5}\ \mathrm{m}^{-3}\mathrm{s}^{-1}$ [4]

SI Table 1 Rates used with literature justification in **Figure 2D.** Note that k_{TTA} has units of $[m]^{-3}[s]^{-1}$.

2. Reaction Scheme Motivation

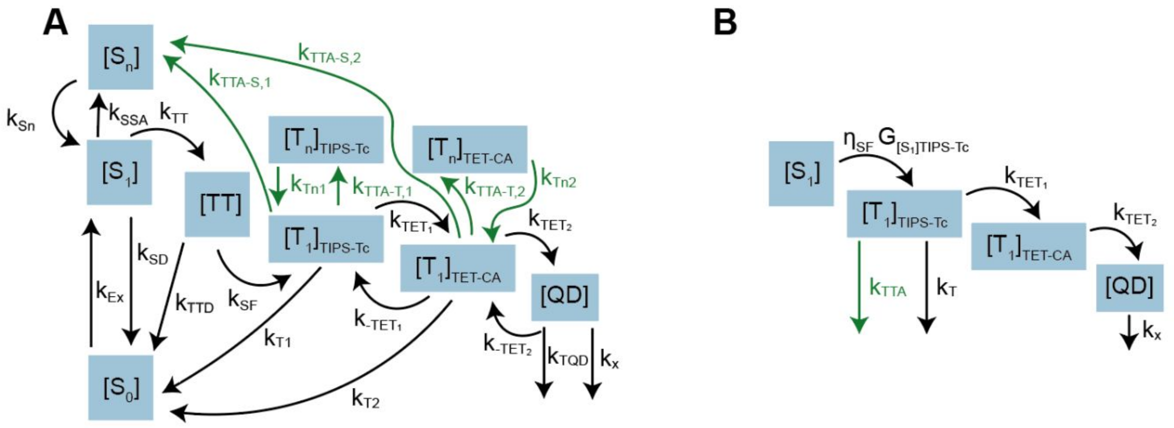

SI Figure 1 A – Reaction scheme following previous approaches in solution [3] [4]. Previous work often utilise literature values from measurements performed in solution with constrained parameters. In films, where we are unable to constrain parameters we use **B** – a reduced reaction scheme to reduce overfitting.

The reduced model used in the main text is represented in **SI Figure 1B,** where we model the transient results in **Figure 3E** following **Equations 1-3** in the main text, reproduced here,

$$(k_T + k_{TET_1})[T_1]_{TIPS-Tc} + k_{TTA}([T_1]_{TIPS-Tc})^2 = \eta_{SF} G_{[S_1]_{TIPS-Tc}}$$

$$-\left(k_{TET_2}\right)[T_1]_{TET-CA} + k_{TET_1}[T_1]_{TIPS-Tc} = 0$$

$$-(k_X)[QD] + \left(k_{TET_2}\right)[T_1]_{TET-CA} = 0$$

Main Text Equation 1-3

A physically more precise model, such as in **SI Figure 1A**, can give rise to an overparametrized problem in the absence of further experiment or constraints. **Main Text Equations 1-3** are similar to analysis regimes previously reported[2,5]. We consider the primary distinction in the reduced model is that the triplet-triplet annihilation pathway directly reduces to the ground state and not to S_n or T_n as in **SI Figure 1A**, rather we treat $\mathrm{k_{TTA}}$ as analogous to a bimolecular decay pathway. **Main Text Equations 1-3** represent the pathways given in **SI Equations 7-11**.

<u>A Triplet Triplet Annihilation Pathway</u>

$$[T_1]_{TIPS-Tc} + [T_1]_{TIPS-Tc} \overset{\mathrm{k_{TTA}}}{\rightarrow} S_0$$

SI Equation 7

We note that some authors scale k_{TTA} by half relative to **Main Text Equation 1-3**[4], typically to account for recovery of some population. Note that our definition of k_{TTA} is a total loss pathway.

A Triplet Triplet Transfer Pathway

$$[T_1]_{TIPS-Tc} \overset{k_{TET_1}}{\rightarrow} [T_1]_{TET-CA}$$

SI Equation 8

A Triplet Decay Pathway

$$[T_1]_{TIPS-Tc} \overset{k_T}{\rightarrow} S_0$$

SI Equation 9

A Triplet to Quantum Dot Transfer Pathway

$$[T_1]_{TET-CA} \overset{k_{TET_2}}{\rightarrow} [QD]$$

SI Equation 10

Quantum Dot Radiative Decay Pathway

$$[QD]^* \overset{k_X}{\rightarrow} [QD] + hv$$

SI Equation 11

A more complete model is represented in the diagram in **SI Figure 1A.** The physical system would be more precisely described by triplet annihilation pathways which regenerate to an excited singlet or triplet, which then decay to the ground state with their own associated rate, k_{S^n} and k_{T^n}, following **SI Equations 12-15**.

Triplet Triplet Annihilation to Singlet

$$T + \mathrm{T} \overset{\mathrm{k_{TTA-S}}}{\rightarrow} S^n + S_0 \overset{k_{\mathrm{sn}}}{\rightarrow} S + S_0$$

SI Equation 12

Recent work suggests that competing pathways can be overcome so that TTA to the singlet exciton can be dominant [6–8], which would reduce these losses, but is practically challenging.

Triplet Triplet Annihilation to Triplet

$$T + T \overset{k_{TTA-T}}{\rightarrow} T^n + S_0 \overset{k_{\mathrm{Tn}}}{\rightarrow} T + S_0$$

SI Equation 13

Further, singlet singlet and singlet triplet annihilation is also possible[9]:

Singlet Triplet Annihilation

$$S + T \overset{k_{STA}}{\rightarrow} T^n + S_0 \overset{k_{T_n}}{\rightarrow} T + S_0$$

SI Equation 14

Singlet Singlet Annihilation

$$S + S \overset{k_{\mathrm{SSA}}}{\rightarrow} S^n + S_0 \overset{k_{\mathrm{Sn}}}{\rightarrow} S + S_0$$

SI Equation 15

We also explicitly discount triplet and singlet annihilation with polarons[9], which is unlikely to be valid in the solid state.

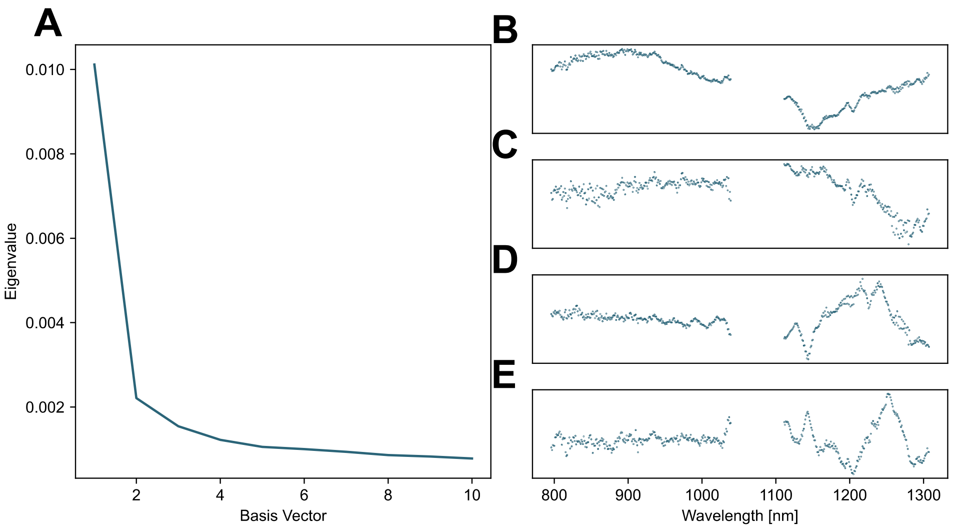

***SI Figure 2 A** - Scree plot of the eigenvalues of the principal components from nsTA data. **B-E** basis vectors 1 through 4 as a function of wavelength. Basis vector 5 has no regular shape.*

To motivate the reduction in dimensionality of the problem, we carried out SVD on the nsTA in **Figure 3E**. SVD can be used to determine the *minimum* number of population components[10] (although SVD itself does not give a direct physical interpretation). Generally, retaining the number of basis vectors whose corresponding singular values have a regular form is a good approximation for the minimal number of bases to retain[11]. In our case, the scree plot (**SI Figure 2**) suggests the presence of 4 basis vectors, which supports our proposed approach to use **Main Text Equations 1-3**.

3. Optimum Concentration

SI Figure 3 depicts TCSPC measurements from films of constant TIPS-Tc loading and varying concentration of PbS-TET-CA under illumination at a constant power of $80\ \mu\mathrm{J/cm^2}$ *at* $400\ \mathrm{nm}$.

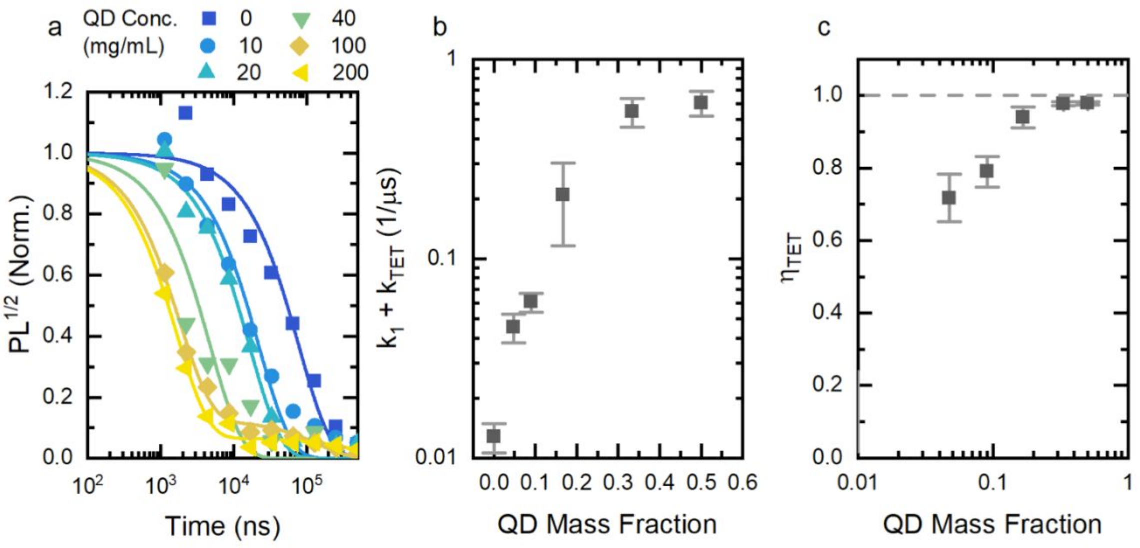

***SI Figure 3** Identification of triplet transfer by monitoring the TIPS-Tc visible PL.*
A - Square root of the singlet component of transient PL from films of TIPS-Tc $(200 mg/mL)$ *and varying amounts of PbS-TET-CA under excitation* $80\mu J/cm^2$ *at* $400\ nm$. *The kinetics have been fit with bi-exponential decays and normalised to the initial value of the decay for clarity. B - Fitted fast decay rate* (k_1) *as a function of the* QD *mass fraction. C - Resulting triplet transfer efficiency based on* k_1 *and an intrinsic triplet decay rate of* $k_T = 0.01\ \mu s^{-1}$.

4. Further Analysis

Main Text Figure 3A&B depict TCSPC measurements from films of constant TIPS-Tc loading and varying concentration of PbS-TET-CA. **SI Figure 4** depicts transient PL measurements of these films. We assign the fast decaying, prompt component (<2 ns lifetime) to the quenching of the singlet emission by singlet fission and the longer delayed component (more than 100 ns after the pump) to long-lived singlet emission resulting from triplet-triplet annihilation back to the singlet state. We distinguish this delayed component from the background detector counts by explicitly measuring the corresponding counts, with no sample under excitation, over the same detector acquisition period. Removal of this background signal allows correction of the PL kinetics followed by normalisation to the value at t=0. With this normalisation it is clear that the addition of PbS-TE-CA QDs to the TIPS- Tc film, results in a decreased delayed PL component. The normalisation of the delayed component by the prompt count rate gives a quantity that is approximately independent of the local density and TIPS-Tc material. Instead, it is only predominately dependent on the local triplet density. As in **Figure 3** the film of pristine TIPS-Tc shows little spatial dependence of the normalised delayed component, indicating a uniform triplet density across the film. However, the PbS-TET-CA:TIPS-Tc film shows variations in the intensity of the TTA intensity on the micron scale. This spatial variation could be the result of spatial variation of the triplet yield from singlet fission, variations in the density of PbS-TET-CA as associated harvesting or differences in the triplet diffusion coefficient. Under the assumption that this spatial variation is due to triplet density variations, the TTA PL suggests that the treatment of magnitude of the singlet emission, due to TTA, as proportional to the square of the triplet density, is appropriate.

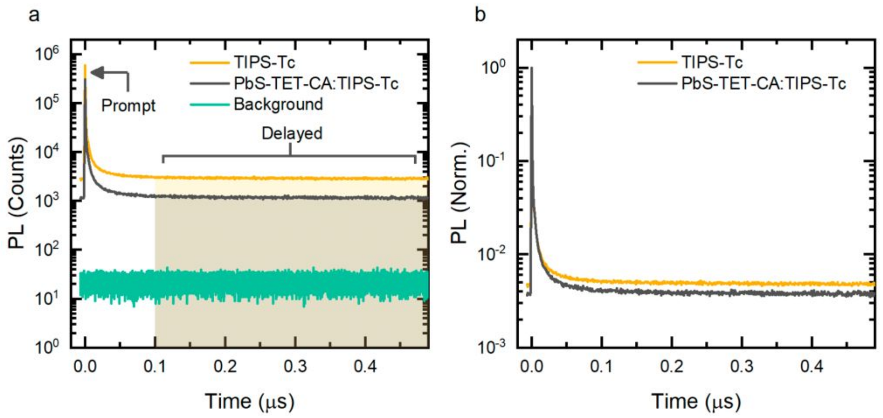

***SI Figure 4**: TIPS-Tc transient visible PL in TIPS-Tc and PbS-TET-CA:TIPS-Tc films. The TIPS-Tc PL decay is detected at* $550 \pm 40\ nm$ *after 405 nm excitation at a fluence of* $50\mu cm^{-2}$ *and 2 MHz pump rep rate. a) Kinetics found by summation of PL decays over entire* $\sim 1000\mu m^2$ *area of investigation. With no sample in the microscope, the background counts over the same integration time was measured (green curve). b) Background corrected and normalised PL kinetics. The TIPS-TC:PbS-TET-CA film was prepared at a 2:1 mass ratio.*

Although not conclusive, we make an attempt to analytically determine the extraction efficiency of triplet excitons as a function of fluence using the TIPS-Tc triplet and QD excited state populations described by **Main Text Equations 1-3**.

For an LSC with optical attenuation coefficient μ the incident light intensity under steady state illumination decays via $I(z) = I_0 e^{-\mu z}$, resulting in a total generation rate $G(z) = G_T(z) + G_{QD}(z)$, given by $-\frac{dI}{dz} = \mu I_0 e^{-\mu z}$. The individual component generations rates are given by, $G_i(z) = \alpha_i G(z)$, where α_i is related to the individual components attenuation coefficients μ_i by

$$\alpha_i = \frac{\mu_i}{\sum_j \mu_j}$$

SI Equation 16

Where we neglect any intermediate state facilitating the transfer between the SF-host and QD. We assume triplet diffusion is relatively slow compared to the spatial variation of the population densities across the thickness of the film, such that we exclude any explicit interaction between spatially adjacent population densities. We approximate the fractional absorption of each component, α_i, as that of the solution phase.[2] From the solid-state TIPS-Tc attenuation coefficient, $\mu_{Tc}(\lambda)$, and the fractional absorption ($\alpha_{Tc}(\lambda)$) from solution phase measurements we calculate the QD attenuation coefficient of the films by,

$$\mu_{QD}(\lambda) = \mu_{Tc}(\lambda)\left(\frac{1}{\alpha_{Tc}(\lambda)} - 1\right)$$

SI Equation 17

Solving for LSC active layer thickness L allows the calculation of the spatial dependence of the photon flux $I(z)$, TIPS-Tc triplet density $[T_1](z)$ and the triplet transfer efficiency $\eta_{\mathrm{TET}}\,(z)$. As in **SI Figure 5**, the inclusion of a bimolecular decay rate reduces the expected triplet population in volumes where the density is high, resulting in a corresponding reduction in the local triplet transfer efficiency.

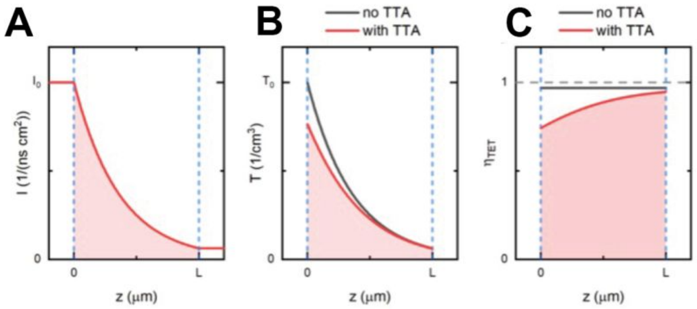

***SI Figure 5** Illustration of the spatial variation in triplet harvesting. **A -** Example calculation of the light intensity **B** - triplet exciton density **C** - triplet exciton transfer efficiency in a SF-LSC as a function of depth into the film.*

To calculate the LSC efficiency under continuous illumination we solve the rate equations **Main Text Equations 1-3** for steady-state conditions, where the system of differential equations reduces to

$$(k_{\mathrm{T}} + k_{TET})\,[T_1] + k_{\mathrm{TTA}}[T_1]^2 = \eta_{SF} \cdot G_{[S_1]_{TIPS-Tc}}\ (z)$$
$$k_x[QD] = k_{TET}[T_1] + G_{QD}(z)$$

SI Equation 18

Solving for positive $[T_1]$ leads to

$$[T_1] = -\frac{k_{\mathrm{T}} + k_{TET}}{2k_{\mathrm{TTA}}} + \sqrt{\left(\frac{k_{\mathrm{T}} + k_{TET}}{2k_{\mathrm{TTA}}}\right)^2 + \frac{\eta_{SF} \cdot G_{[S_1]_{TIPS-Tc}}\ (z)}{k_{\mathrm{TTA}}}}.$$

SI Equation 19

The triplet transfer efficiency is given by $\eta_{TET}(z) = \frac{k_{TET}[T_1]}{\eta_{SF} \cdot G_{[S_1]_{TIPS-Tc}}(z)}$. Substituting the above expression for $[T_1]$ gives

$$\eta_{TET}(\mathrm{z}) = \frac{k_{TET}}{\eta_{SF} \cdot G_{[S_1]_{TIPS-Tc}}\ (z)}\left(\sqrt{\left(\frac{k_{\mathrm{T}} + k_{TET}}{2k_{\mathrm{TTA}}}\right)^2 + \frac{\eta_{SF} \cdot G_{[S_1]_{TIPS-Tc}}\ (z)}{k_2}} - \frac{k_{\mathrm{T}} + k_{TET}}{2k_{\mathrm{TTA}}}\right)$$

SI Equation 20

Then substituting the value for $G_T(z)$ gives

$$\eta_{TET}(\mathrm{z}) = \frac{k_{TET}}{\eta_{SF}\cdot\alpha_T G(z)}\left(\sqrt{\left(\frac{k_\mathrm{T}+k_{TET}}{2k_\mathrm{TTA}}\right)^2+\frac{\eta_{SF}\cdot\alpha_T G(z)}{k_\mathrm{TTA}}}-\frac{k_\mathrm{T}+k_{TET}}{2k_\mathrm{TTA}}\right)$$

SI Equation 21

The overall photomultiplier efficiency is

$$\eta_{PM}(z) = \eta_{QD}\big(\alpha_{QD}+\alpha_T\eta_{SF}\eta_{TET}(z)\big).$$

SI Equation 22

The rate of QD emission in the LSC can be expressed as

$$PL_{QD} = \int_0^L \eta_\Omega G(z)\eta_{PM}(z)dz,$$

SI Equation 23

where η_Ω represents the PL collection and detector efficiency. Substituting the relevant expression leads to

$$PL_{QD} = \int_0^L \eta_\Omega\eta_{QD}\left(\alpha_{QD}G(z)+k_{TET}\left(\sqrt{\left(\frac{k_\mathrm{T}+k_{TET}}{2k_\mathrm{TTA}}\right)^2+\frac{\eta_{SF}\cdot\alpha_T G(z)}{k_\mathrm{TTA}}}-\frac{k_\mathrm{T}+k_{TET}}{2k_\mathrm{TTA}}\right)\right)dz$$

SI Equation 24

Substituting $G(z)=\mu I_0 e^{-\mu z}$ gives

$$PL_{QD} = \int_0^L \eta_\Omega\eta_{QD}\left(\alpha_{QD}\mu I_0 e^{-\mu z}+k_{TET}\left(\sqrt{\left(\frac{k_\mathrm{T}+k_{TET}}{2k_\mathrm{TTA}}\right)^2+\frac{\eta_{SF}\cdot\alpha_T\mu I_0 e^{-\mu z}}{k_\mathrm{TTA}}}-\frac{k_\mathrm{T}+k_{TET}}{2k_\mathrm{TTA}}\right)\right)dz$$

SI Equation 25

SI Equation 24 has an analytical solution, specifically, the integral $\int_0^U\sqrt{Y^2+X\mathrm{e}^{-Jz}}-Ydz$ has the analytical solution

$$\frac{\sqrt{Xe^{-JU}+Y^2}\left(-2\sqrt{Y^2e^{JU}+X}+2Ye^{\frac{JU}{2}}\log\left(Y\left(e^{-\frac{JU}{2}}\sqrt{Y^2e^{JU}+X}+Y\right)\right)+JUYe^{\frac{JU}{2}}\right)}{J\sqrt{Y^2e^{JU}+X}}$$
$$+2\,\frac{\left(\sqrt{X+Y^2}-Y\log\left(Y(\sqrt{X+Y^2}+Y)\right)\right)}{J}-UY.$$

SI Equation 26

SI Equation 25 can then be solved for any given set of system parameters, including for arbitrary excitation wavelength and incident fluence. The efficiency of device will be dependent on the absorption of tetracene, as a reduced triplet population will have proportionally smaller k_{TTA} loss. Using excitation spectra of PbS-TET-CA:TIPS-Tc under solar-equivalent fluence, we found the the PbS-TET-CA:TIPS-Tc film's excitation spectrum matches with the simulated spectrum, see **SI Figure 6B** for the kinetic parameters detailed in **SI Table 2** and a triplet bimolecular decay rate of $\mathrm{k_{TTA}} = 1 \times 10^{-18}\ \ \mathrm{cm^3\ ns^{-1}}$. We could therefore quantify the gain if k_{TTA} was reduced. As expected, the gains are felt most strongly in the regions the tetracene is strongly absorbing. We can expect different efficiencies depending on where the tetracene is excited.

The LSC device efficiency[12] can then be calculated from the PL rate PL_{QD} (units counts per second) by accounting for laser/flux input and normalising for photons to give $PL_{QD,n}$ (dimensionless),

$$\eta_{\mathrm{dev}}\,(G) = \frac{\overbrace{V_{\mathrm{OC}}\,\mathrm{FF}\int_0^\infty \lambda/hc\ \mathrm{AM1.5}(\lambda)\alpha(\lambda)\mathrm{d}\lambda \int_0^\infty q\eta_{\mathrm{int}}\,(G)PL_{QD,n}(\lambda)\mathrm{EQE}\,(\lambda)\mathrm{d}\lambda}^{\propto I_{\mathrm{SC}}}}{\int_0^\infty \mathrm{AM1.5}(\lambda)\mathrm{d}\lambda}$$

SI Equation 27

where q is the electronic charge, $\eta_{int}(G)$ is the LSC internal optical efficiency as a function of LSC size and we may approximate $PL_{QD,n}(G,\lambda) = PL_{QD,n}(\lambda)$ since we observe negligible reabsorption losses (see **Figure 4B**). **SI Equation 27** is used for **Figure 4C.**

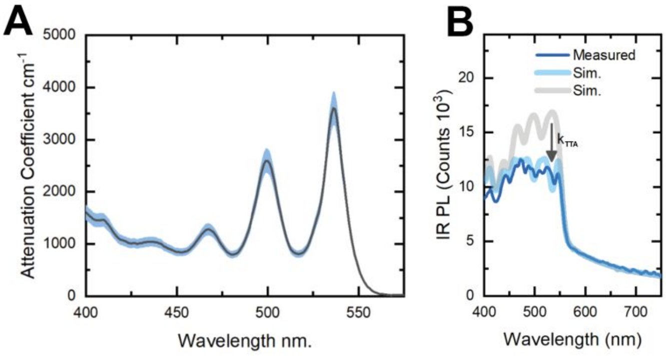

***SI Figure 6 A -** Thin-film TIPS-Tc attenuation coefficient spectrum (black curve) from a 220 ± 20 nm thick film of TIPS-Tc. The uncertainty in the attenuation coefficient is calculated from the uncertainty in the film thickness (light blue). **B** - Excitation spectra of PbS-TET-CA:TIPS-Tc under solar-equivalent fluence. The PbS-TET-*

*CA:TIPS-Tc film's excitation spectrum matches with the simulated spectrum (**SI Equation 25**)for kinetic parameters detailed in **SI Table 2** and a triplet bimolecular decay rate of $k_{TTA} = 1 \times 10^{-18}\ \ cm^3\ ns^{-1}$ (light blue curve). The theoretical increased dependence light grey curve is given for the low k_{TTA}→$10^{-21} cm^{-3} ns^{-1}$ regime.*

We model **SI Equation 27,** specifically $V_{\mathrm{OC}}\ \mathrm{FF}\ , EQE\ (\lambda)$ following the approach we previously reported for silicon solar cells[13]. Briefly, we characterise the cell as a set of diodes, where each form of carrier recombination contributes to a loss in current density, $J(V)$, with units of amps per metre squared, at a given voltage, V. The equation,

$$J(V) = J_G(EQE,\Gamma) - J_{Rad}(V,R_S,T) - J_{nRad}(V,R_S,T) - J_{\mathrm{Auger}}\ (V,R_S,L,T) - \frac{V + JR_S}{R_{\mathrm{Shunt}}}$$

SI Equation 28

describes this relationship, where $J(V)$ measured current density, J_{Auger} is the auger recombination current density, J_{Rad} is the radiative recombination current density, J_{nRad} is non-radiative recombination, and $\frac{v+JR_{\mathrm{Series}}}{R_{\mathrm{Shunt}}}$ is the current lost due to parasitic shunt resistance (R_{Shunt}). The expressions for J_{Auger} , J_{Rad} , J_{nRad} and R_{Shunt} can be found elsewhere[13]. Taking a best-in-class Si solar cell[14], with LSC $G \approx 15$, and our measured optical efficiency of $\eta_{int}(15) = 13\%$, as we have here, we may now model the impact of PL overlap with the EQE and k_{TTA} rate.

Code to reproduce **Figure 4C** is provided at GitHub repository (Tb8854/SF-LSC-Paper).

Parameter	Value	Comment
Solid State Attenuation μ_T	**SI Figure 6A**	
Fractional Absorption, α_i	**Main Text Figure 2C**	Determined from solution.
$\eta_\Omega\eta_{QD}$	9300 counts	Conversion factor between absorption and detector counts. Depends on detector configuration. Can be normalised by dividing by absorption spectra to give relative PLQE.
L	50 μm	Blade coating & parameter fit.
η_{SF}	190%	[15]
$\eta_{\mathrm{TT}}\ \eta_{\mathrm{TD}}$	97%	[15]
η_{PL}	30%	Determined from toluene solution. [15]
PL_{QD}	17%	This work.
k_{TTA}	$[10^{-18} - 10^{-21}]\ \mathrm{cm^{-3}ns^{-1}}$	This work, 10^{-18} determined from fitting to nsTA results and larger values from terrestrially acceptable LSC devices (**Figure 3**)
k_{TET}	0.3 μs^{-1}	Solid state, see main text.
k_T	0.01 μs^{-1}	Solid state, see main text.
η_{dev}	<0.01%	This work.
η_{int}	13%	This work

SI Table 2 Summary of parameters used for simulation.

Bibliography

1. Wilson, M. W. B. *et al.* Temperature-independent singlet exciton fission in tetracene. *J Am Chem Soc* **135**, 16680–16688 (2013).
2. Allardice, J. R. *et al.* Engineering Molecular Ligand Shells on Quantum Dots for Quantitative Harvesting of Triplet Excitons Generated by Singlet Fission. *J Am Chem Soc* **141**, 12907–12915 (2019).
3. Gray, V. *et al.* Direct vs Delayed Triplet Energy Transfer from Organic Semiconductors to Quantum Dots and Implications for Luminescent Harvesting of Triplet Excitons. *ACS Nano* **14**, 36 (2020).
4. Daiber, B. *et al.* Change in Tetracene Polymorphism Facilitates Triplet Transfer in Singlet Fission-Sensitized Silicon Solar Cells. *Journal of Physical Chemistry Letters* **11**, 8703–8709 (2020).
5. Lavarda, G. *et al.* Long-Lived Triplets from Singlet Fission in Pentacene-Decorated Helical Supramolecular Polymers. *J Am Chem Soc* **146**, 28985–28993 (2024).
6. Cheng, Y. Y. *et al.* Kinetic analysis of photochemical upconversion by triplet-triplet annihilation: Beyond any spin statistical limit. *Journal of Physical Chemistry Letters* **1**, 1795–1799 (2010).
7. Di, D. *et al.* Efficient Triplet Exciton Fusion in Molecularly Doped Polymer Light-Emitting Diodes. *Advanced Materials* **29**, 1605987 (2017).
8. Hoseinkhani, S., Tubino, R., Meinardi, F. & Monguzzi, A. Achieving the photon up-conversion thermodynamic yield upper limit by sensitized triplet–triplet annihilation. *Physical Chemistry Chemical Physics* **17**, 4020–4024 (2015).
9. Forrest, S. R. Organic electronics: Foundations to applications. *Organic Electronics: Foundations to Applications* 1–1014 (2020) doi:10.1093/oso/9780198529729.001.0001.
10. Ruckebusch, C., Sliwa, M., Pernot, P., de Juan, A. & Tauler, R. Comprehensive data analysis of femtosecond transient absorption spectra: A review. *Journal of Photochemistry and Photobiology C: Photochemistry Reviews* **13**, 1–27 (2012).
11. Dorlhiac, G. F., Fare, C. & van Thor, J. J. PyLDM - An open source package for lifetime density analysis of time-resolved spectroscopic data. *PLoS Comput Biol* **13**, e1005528 (2017).
12. Baikie, T. K., Xiao, J., Drummond, B. H., Greenham, N. C. & Rao, A. Spatially Resolved Optical Efficiency Measurements of Luminescent Solar Concentrators. *ACS Photonics* (2023) doi:10.1021/ACSPHOTONICS.3C00601.
13. Baikie, T. K. *et al.* Revealing the potential of luminescent solar concentrators in real-world environments. *Joule* **8**, 799–816 (2024).
14. Green, M. A. *et al.* Solar cell efficiency tables (version 50). *Progress in Photovoltaics: Research and Applications* **25**, 668–676 (2017).
15. Gray, V. *et al.* Ligand-Directed Self-Assembly of Organic-Semiconductor/Quantum-Dot Blend Films Enables Efficient Triplet Exciton-Photon Conversion. *J Am Chem Soc* **146**, 7763–7770 (2024).
